# Targeting *S1PR1* May Result in Enhanced Migration of Cancer Cells in Bladder Carcinoma

**DOI:** 10.3390/cancers13174474

**Published:** 2021-09-05

**Authors:** Chin-Li Chen, En Meng, Sheng-Tang Wu, Hsing-Fan Lai, Yi-Shan Lu, Ming-Hsin Yang, Chih-Wei Tsao, Chien-Chang Kao, Yi-Lin Chiu

**Affiliations:** 1 Division of Urology, Department of Surgery, Tri-Service General Hospital, National Defense Medical Center, Taipei 11490, Taiwan; iloveyou@mail.ndmctsgh.edu.tw (C.-L.C.); mengen@mail.ndmctsgh.edu.tw (E.M.); doc20283@mail.ndmctsgh.edu.tw (S.-T.W.); yangming@mail.ndmctsgh.edu.tw (M.-H.Y.); wei@mail.ndmctsgh.edu.tw (C.-W.T.); everyyoung@mail.ndmctsgh.edu.tw (C.-C.K.); 2 Department of Biochemistry, National Defense Medical Center, Taipei 11490, Taiwan; 809302001@mail.ndmctsgh.edu.tw (H.-F.L.); 608040006@mail.ndmctsgh.edu.tw (Y.-S.L.)

**Keywords:** sphingosine 1-phosphate receptor 1, bladder carcinoma, cell migration, epithelial–mesenchymal transition, FTY-720

## Abstract

**Simple Summary:**

Metastasis is critical to the prognosis of patients with bladder cancer, and it is important to understand the mechanism of its occurrence. *S1PR1* expression is thought to be associated with poor prognosis, but it is unknown whether it is associated with tumor metastasis. Analysis of clinical gene expression data suggests that endothelial or immune cells in tumor tissue may be the source of *S1PR1* expression. Comparative analysis of clinical tumor tissues with bladder cancer cells suggests that *S1PR1* expression is associated with cellular adhesion. In vitro experiments demonstrated that *S1PR1* expression was negatively correlated with cancer cell motility, and that *S1PR1* inhibition by FTY-720 may cause an increase in cancer cell motility, suggesting that the use of *S1PR1* inhibition as a synergistic therapy requires additional observations and considerations.

**Abstract:**

Clinical bladder tumor histological analysis shows that high expression of *S1PR1* is associated with poor patient prognosis. However, there are no studies that describe the underlying mechanism. To investigate the relative distribution and actual function of *S1PR1* in bladder tumors, we analyzed multiple clinical databases in combination with tumor purity and immune cell infiltration simulations, as well as databases of well-defined histological phenotypes of bladder cancer, and single-cell sequencing of adjacent normal tissues and bladder tumors, and further compared them with bladder cancer cell lines. The results showed that **S1PR1** expression was generally higher in normal tissues than in bladder cancer tissues, and its distribution was mainly in endothelial cells or immune cells. The association between high **S1PR1** expression and poor prognosis may be due to tumor invasion of adjacent normal tissues, where highly expressed **S1PR1** may affect prognostic interpretation. The effect of *S1PR1* itself on cancer cells was associated with cell adhesion, and in bladder cancer cells, *S1PR1* expression was negatively correlated with cell motility. Moreover, the use of FTY-720 will cause an increased metastatic ability of bladder cancer cells. In conclusion, we suggest that the use of *S1PR1*-specific inhibition as a synergistic treatment requires more observation and consideration.

## 1. Introduction

Urothelial carcinoma of the bladder (UCB) is among the top 10 most common cancers in the world, with an estimated 80,000 new cases and 17,000 deaths in the United States each year [1,2,3]. Significant advances have been made in the management of bladder cancer since the 1990s. More accurate staging has been achieved with refined tissue imaging, and advances in surgical techniques have been combined with improved chemotherapy regimens. Even more, the 5-year survival rate for patients with non-muscle invasive UCB is over 90% [1], and radical cystectomy is the treatment of choice for patients with surgically resectable disease without evidence of metastatic disease. However, patients with muscle-invasive bladder cancer or disseminated disease have a much lower survival rate [4,5], suggesting that the occurrence of metastasis has a significant impact on the prognosis of patients with bladder cancer. Considering the impact of metastatic disease on treatment options and patient prognosis, the importance of timely detection and prevention of metastasis in UCB cannot be overemphasized.

Sphingosine 1-phosphate receptor 1 (*S1PR1*) is a biologically active sphingolipid metabolite receptor, whose ligand Sphingosine 1-phosphate (S1P) is known to modulate cell survival, migration, immune cell transport, angiogenesis, and vascular barrier function [6]. Physiologically, *S1PR1*, which is abundantly expressed in vascular endothelial cells (EC), is important for embryonic vascular development and maturation [6]. In addition, *S1PR1* expression in immune cells is thought to be associated with the regulation of traffic between tissues, including B cells, T cells, natural killer cells, macrophage, monocyte, and neutrophil [7,8,9,10,11,12]. In cancer progression, *S1PR1* is thought to be highly expressed in bladder cancer cells and is associated with poor patient prognosis [13]. S1P can promote cancer cell viability, survival, growth, and transformation by activating *S1PR1* [14]. In addition, **S1PR1** overexpression is associated with the convening of regulatory T cells (T_reg_) [15], suggesting **S1PR1** as a potential prognostic biomarker and therapeutic target for UCB patients. Clinically, FTY-720 (Fingolimod) is used as an *S1PR1* inhibitor and is widely used in multiple sclerosis or as an immunomodulator [16,17]. FTY-720 is reported to promote apoptosis of bladder cancer cells [18]. As *S1PR1* is important for the regulation of immune cell movement, we wondered whether it might have a similar role in the metastasis of bladder cancer cells. However, given the complexity of tumor tissue composition, such as endothelial cells and immune cells, which are rich in *S1PR1* expression, it is still unknown whether *S1PR1* is actually overexpressed in bladder cancer cells or whether *S1PR1* expression has a substantial effect on bladder cancer cells themselves.

In this study, we analyze the association between **S1PR1** expression and patient prognosis by exploring several bladder cancer clinical databases. In addition, the association of **S1PR1** with tumor purity and immune cell infiltration was comprehensively analyzed. The main distribution of **S1PR1** in bladder tumors was analyzed in microarray and single cell sequencing databases with well-defined histological patterns. Using comparative analysis of bladder cancer cell lines and clinical tissues, we confirmed the association of *S1PR1* with cell adhesion ability in bladder cancer cells. A negative correlation between *S1PR1* expression and cell motility was also confirmed in bladder cancer cell lines. Lastly, the effect of FTY-720 on promoting bladder cancer cell motility was confirmed in cell lines and patient-derived tumor cultures. This article first revealed that loss of **S1PR1** expression in bladder cancer cells is associated with increased cell motility and that inhibition of *S1PR1* activity with FTY-720 may cause a similar phenomenon. We suggest that the use of FTY-720 as a synergistic strategy for other treatments requires more evaluation and observation.

## 2. Materials and Methods

### 2.1. Patient Specimens and Clinical Data

The bladder tissue obtained for the study was obtained in accordance with the relevant guidelines and regulations and was approved by the Institutional Review Board of Tri-service General Hospital, National Defense Medical Center (IRB approval ID:1-108-05-130). Tumors were obtained from patients who underwent transurethral resection of bladder tumor (TURBT) and signed an informed consent form. Patient information and related clinical information were de-identified.

### 2.2. Gene Expression Database Collection and Analysis

We searched the NCBI-GEO database (http://www.ncbi.nlm.nih.gov/geo/, accessed on 18 June 2018) for gene expression studies related to urothelial carcinoma of the bladder using the keyword “urothelial carcinoma”. The following criteria were used for selection: (1) datasets included **S1PR1** gene probes; (2) had overall survival status and survival time; and (3) for each bladder urothelial cancer tissue dataset, the total number of available samples was greater than 40. To investigate the prognostic relevance of **S1PR1** grouping without a predetermined stance, we used Evaluate Cutpoints [19] to define the best cut-points in terms of **S1PR1** mRNA expression, survival status, and survival time. Meta-analyses were performed on TCGA BLCA (The Cancer Genome Atlas-Bladder Urothelial Carcinoma) (424 patients), GSE5287 (49 patients), GSE13507 (164 patients), GSE31684 (93 patients), GSE32894 (307 patients), and GSE48277 (159 patients).

For the expression of **S1PR1** in different cancer types in TCGA, we searched for “**S1PR1**” in “Gene DE” in the “Exploration” tab of TIMER2.0 [20]. For the single cell sequencing database of bladder cancer published by Chen et al., we analyzed and plotted the distribution of **S1PR1** expression in R using the scripts provided by the authors. The distribution of tumor grade and cell type was directly quoted from the authors’ publication [21]. Cancer cell line encyclopedia (CCLE) and TCGA BLCA whole gene expression matrix (FPKM) with corresponding clinical parameters for patients or methylation expression profiles and copy number for cell lines were downloaded from UCSC XENA (https://xenabrowser.net/, accessed on 26 March 2021) [22]. The hierarchical cluster function provided in Morpheus (Broad Institute, https://software.broadinstitute.org/morpheus/, accessed on 4 March 2021) was used to cluster **S1PR1** expression, methylation, and copy number data obtained from the Cancer Cell Line Encyclopedia (CCLE) database [23].

### 2.3. Evaluation of Tumor Purity and Immune Cell Simulated Infiltration

For the obtained database, we used the ESTIMATE R software package to calculate the ESTIMATE scores and used the formula of Yoshihara et al. to calculate the purity [24]. The ESTIMATE scores of TCGA BLCA were downloaded directly from the authors’ website (https://bioinformatics.mdanderson.org/estimate/disease.html, accessed on 26 March 2021). For the in silico simulation of immune cell infiltration analysis, we used the quanTIseq function provided in immunedeconv and set the parameters according to the authors’ instructions to adapt to different types of databases [25,26]. For *S1PR1* gene expression and purity and multiple immune cell infiltration scores, we calculated Spearman’s correlation and *p*-values, and graphed the correlation matrix with OriginPro 2021b (Origin Lab, Northampton, MA, USA).

### 2.4. Enrichment Map Visualization

The rationale and operation were performed as previously described [27,28]. Briefly, the TCGA BLCA and GSE13507 databases were chosen based on (1) the largest number of patient samples and (2) the divergence of *S1PR1* expression and prognosis. We used the BP:GO bioprocess (7530 gene sets) downloaded from Molecular Signatures Database (MsigDB) for gene-set enrichment analysis (GSEA). Samples were categorized as “**S1PR1** high vs. **S1PR1** low” according to the annotation elsewhere in this study. All nodes presented have passed the screening of |NES| > 1.5, FDR < 0.01 (NES: normalized enrichment score, FDR: false discovery rate).

### 2.5. Messenger RNA Expression Analysis

Total RNA from tumor and adjacent tissues or cultured cells was isolated using a Qiagen RNeasy Mini Kit (Qiagen, Hilden, Germany). RNA concentration and quality were assessed using SpectraMax iD3 (Molecular Devices, San Jose, CA, USA). The cDNA was generated from 2 μg of RNA by reverse transcription using MMLV high performance reverse transcriptase (Epicentre Technologies, Madison, WI, USA) according to the manufacturer’s instructions. Real-time PCR was performed using an CFX96 Touch Real-Time PCR Detection System (Bio-Rad, Hercules, CA, USA). The cycling condition was 95 °C for 12 min, followed by 40 cycles of 95 °C for 15 s, 60 °C for 20 s, and 72 °C for 20 s. The housekeeping gene Glyceraldehyde 3-phosphate dehydrogenase (GAPDH) was measured as an internal control. The expression level of target genes was analyzed by the relative quantity (RQ) value calculated using the ΔΔCt method [Δ(Ct_TARGET_ − Ct_GAPDH_)_sample_ − Δ(Ct_TARGET_ − Ct_GAPDH_)_calibrator_] in triplicate. All primer sequences are listed in the following Table 1.

### 2.6. Immunoblotting

The bladder cancer cell pellets or pretreated tumor specimens were washed twice with phosphate-buffered saline (PBS), lysed in radioimmunoprecipitation assay (RIPA) buffer, and quantified by Bradford protein assay (Bio-Rad, Hercules, CA, USA; #500-0006). Firstly, 30–50 μg of quantified total protein lysate was loaded into each well of the gel, analyzed in 8–10% SDS-PAGE under reducing conditions, and then transferred to a nitrocellulose blotting membrane (PALL Corpo., Pansacola, FL, USA) followed by blocking in 5% skim milk. The membrane was stained with primary antibody as follows: *S1PR1*(Abclonal Inc., Woburn, MA, USA; A3997); E-cadherin (Cell signaling, Danvers, MA, USA; #3195); Vimentin (Abclonal Inc., Woburn, MA, USA; A11952); N-cadherin (Cell signaling #4061); Fibronectin (Finetest, Wuhan, China; fnab03122); SNAI1 (Cell signaling, #3879); Slug (Cell signaling, #9585); and internal control GAPDH (Cell signaling, #5174), prepared in 1% bovine serum albumin (BSA) in Tris-buffered saline and 0.1% Tween^®^20 (TBST) at 4 °C overnight. Then, the membrane was washed and incubated with secondary antibody at room temperature for 1 h. Signals were detected for 1–10 min using an enhanced chemiluminescence solution (Advansta, Menlo Park, CA, USA) and iBright FL1500 Imaging System (Thermo Fisher Scientific., Waltham, MA, USA). All experiments were performed in duplicate.

### 2.7. Cell Culture and Establishment of Stably Expressed shRNA Cell Lines

The J82 human bladder cancer cell line was purchased from Bioresource Collection and Research Center (BCRC, Hsinchu, Taiwan). Cells were cultured in Dulbecco’s modified Eagle’s medium and supplemented with 10% fetal bovine serum (FBS, Thermo Fisher Scientific) under a humidified atmosphere of 5% CO_2_ at 37 °C. For the subcultures, cells were trypsinized with 0.05% Trypsin-EDTA (Thermo Fisher Scientific).

All the short hairpin RNA (shRNA) clones were obtained from National RNAi Core Facility (Genomics Research Center, Academia Sinica, Taipei, Taiwan). The shRNA against **S1PR1** target sequence was 5′-GACAACCCAGAGACCATTATG-3′ (clone ID: TRCN0000356960, sh*S1PR1*#1), 5′-CCCATGTGAAAGCGTCTCTTT-3′ (clone ID: TRCN0000221119, sh*S1PR1*#2), and 5′- TCCTAAGGTTAAGTCGCCCTCG-3′ (clone ID: TRCN0000072249, shLuc) for firefly luciferase as the negative control. The shRNA plasmids were transfected into J82 cell lines with Lipofectamine 3000 (Thermo Fisher Scientific.) according to the manufacturer’s instructions. The stably expressed shRNA cell lines were established with the screening of puromycin 2 μg/mL for 1 week. Knockdown efficiency of **S1PR1** was confirmed by quantitative reverse transcriptase-PCR (data not shown) and Western blot analysis at 24 to 48 h post-transfection (Uncropped Western blot images were provided in Supplementary File S1).

### 2.8. Wound Healing Assay and LIVE Cell Imaging

Migration was evaluated in duplicate by seeding cells on both sides of an Ibidi culture insert (Ibidi, Munich, Germany) with a 500 µm separation gap. J82 shLuc bladder cancer cell line transfected with **S1PR1**/pcDNA 3.1(+) (1 or 3 μg) or pcDNA 3.1(+) (3 μg) ws grown for 24 h, then the growth medium was changed for complete RPMI1640 supplied with 0.5% FBS for 24 h before wound healing assay to diminish the potential interfere of cell proliferation. The gaps of J82 cells were time-lapse photographed every 30 min for 24 h and 48 h using Lumascope 620 with a 10× objective (Etaluma, San Diego, CA, USA), and all experiments were performed in duplicate. For multi-dose FTY-720 (MedChemexpress, Monmouth Junction, NJ, USA; HY-12005) treatment gap healing analysis, we used the ImageXpress Pico system (Molecular Devices) to detect hourly changes in the total number of cells in the gap over a 48 h period. Each dose was duplicated, and the curve results were presented as mean only to minimize interpretation interference. Migration ability of cancer cells was evaluated by Chemotaxis and Migration Tool 2.0 (Ibidi) and Manual Tracking plug-in in ImageJ 1.53h (National Institutes of Health, Stapleton, NY, USA).

### 2.9. Transwell Migration Assay

For the Transwell migration assay, cells were collected and transfected at a density of 10^5^/well for 24 h. Cells were placed in 200 μL of serum-free medium and inoculated in the upper compartment of the chamber. Then, 1 mL of complete medium containing 10% FBS was added to the lower compartment. After 24 h of incubation, the chambers were removed and the cells on the upper surface of the membrane were wiped off with a cotton swab. Then, the cells invading the microporous membrane were washed three times with PBS, fixed with 4% paraformaldehyde solution for 30 min, and stained with 0.1% crystal violet (Sigma-Aldrich, St. Louis, MO, USA) for 20 min. Finally, the cells were observed with a microscope (CKX53, Olympus, Tokyo, Japan) and images were taken for further imageJ analysis. All experiments were performed in duplicate.

### 2.10. Patient-Derived Tumor Primary Culture (PDC)

The experimental procedure is based on the publication of van de Merbel et al. with minor modifications [29]. Briefly, freshly collected bladder tissues were divided into multiple 1 mm^3^ slices, and the sliced samples were evenly divided into four aliquots and collected for analysis after 48 h of incubation at different concentrations of FTY-720.

### 2.11. Statistical Analysis

GraphPad Prism 9.1.2 (GraphPad Software, San Diego, CA, USA) or OriginPro 2021b was used for data analysis and graph production. Student’s t-test was used for analysis of measurement data between two groups, and one-way analysis of variance (ANOVA) was used for multiple group comparisons. Survival curves were plotted using Kaplan–Meier analysis and logarithmic tests. The univariate Cox proportional hazards regression model was used to estimate the odds ratio (OR). Data are expressed as mean ± standard deviation (SD) and all experiments were repeated in duplicate independently at least.

## 3. Results

### 3.1. Retrospective Evaluation of the Association between S1PR1 Expression and Bladder Cancer Prognosis Shows Divergent Results in Different Databases

**Figure 1:**
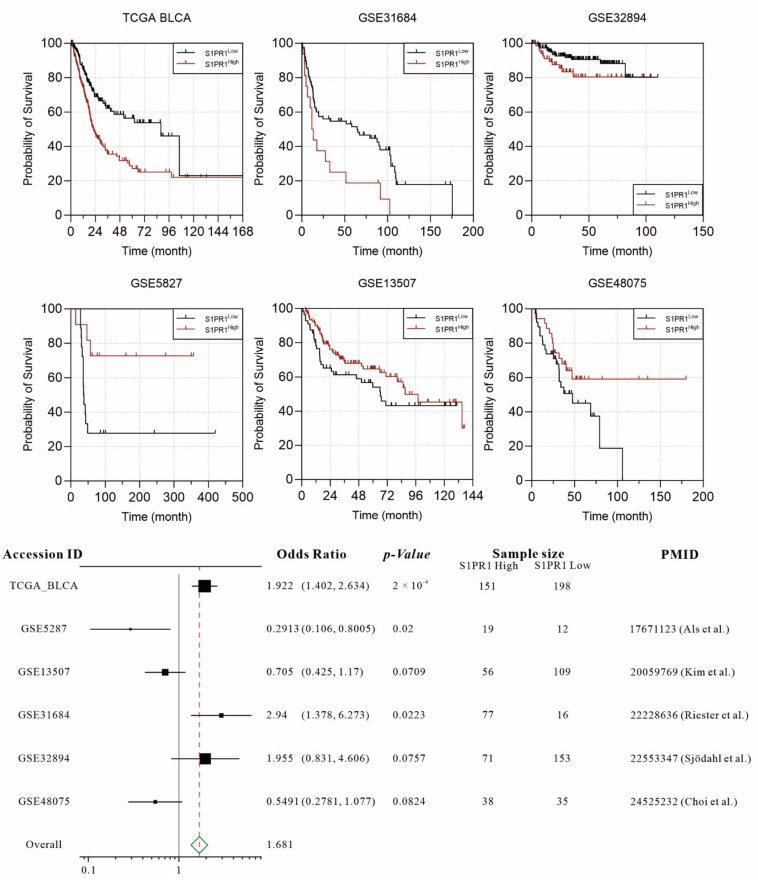
Individual and integrated analysis of the association between *S1PR1* mRNA expression and patient prognosis in published bladder cancer clinical databases [30,31,32,33,34].

Several publications have demonstrated the association of *S1PR1* overexpression with worse prognosis in bladder cancer patients. To comprehensively evaluate the association between *S1PR1* expression and prognosis of bladder cancer patients, we collected six available databases including GSE5287, GSE13507, GSE31684, GSE32894, GSE48075, and TCGA BLCA. In order to unbiasedly group the *S1PR* expression, the “Evaluate Cutpoints” application in R was used to find the best cut-off point for the lowest *p*-value in survival of *S1PR1* expression [19]. The results showed that high *S1PR1* expression in TCGA BLCA (OR: 1.922, *p*-value: 0.0002), GSE 32894 (OR: 1.955, *p*-value: 0.0757), and GSE31684 (OR: 2.94, *p*-value: 0.0223) was associated with worse prognosis. On the contrary, high *S1PR1* expression was associated with better prognosis in GSE5287 (OR: 0.2913, *p*-value: 0.02), GSE48075 (OR: 0.5491, *p*-value: 0.0824), and GSE13507 (OR: 0.705, *p*-value: 0.0709) (Figure 1). The meta-analysis showed that the overall odds ratio still reached 1.681, indicating that, in general, high *S1PR1* expression was associated with a worse prognosis in bladder cancer.

### 3.2. Differences in *S1PR1* Expression and Prognosis of Patients with Bladder Cancer May Be Related to the Degree of Neutrophil Infiltration

**Figure 2:**
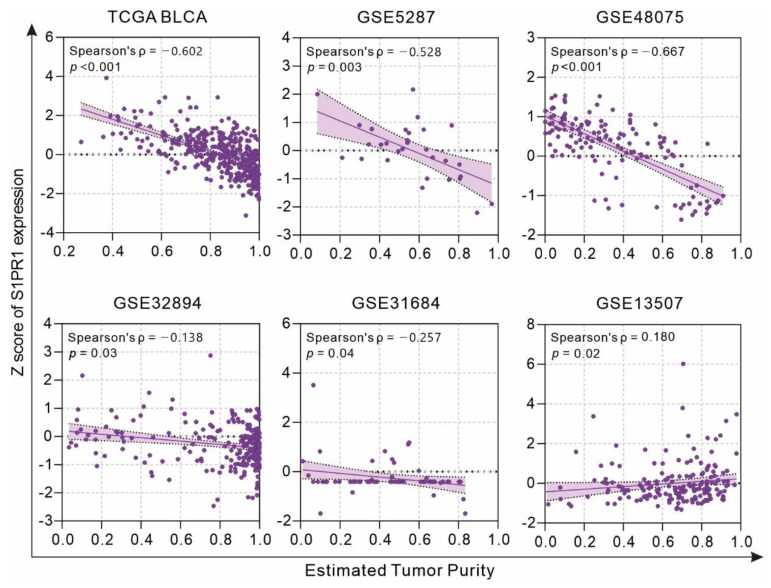
Association of *S1PR1* expression with tumor purity.

Considering that *S1PR1* is an important receptor related to the regulation of migration by various immune cells [12], clinical specimens may have different levels of immune cell infiltration affecting the *S1PR1* mRNA expression in bulky tumors. Evaluation of the association between tumor purity and *S1PR1* expression using the estimate score strategy showed that *S1PR1* expression was negatively correlated with tumor purity in all databases except GSE13507 (Figure 2), suggesting that high *S1PR1* expression in bulk tissue may be associated with enriched immune or stromal cell infiltration.

**Figure 3:**
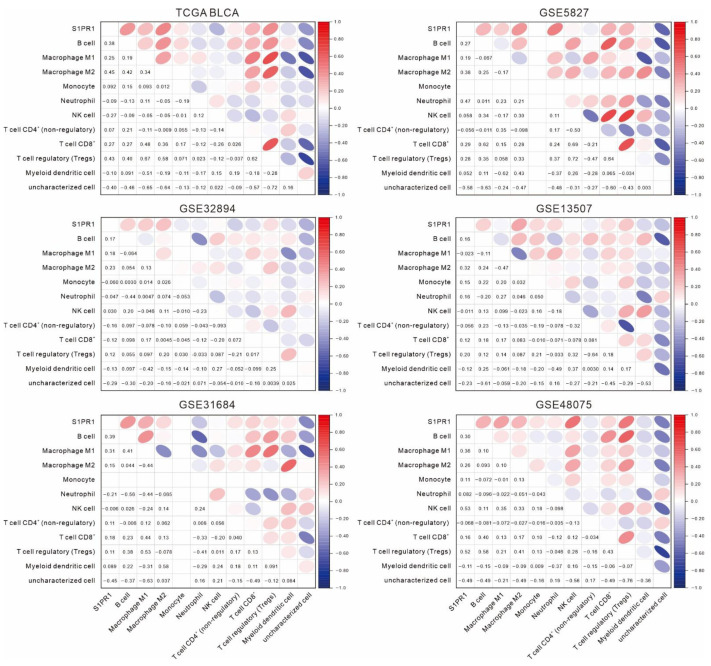
Association of *S1PR1* expression with various immune cell infiltrations. The association of *S1PR1* expression in bladder cancer tissues with QUANTISEQ simulated multiple immune cell infiltration was assessed using Spearman’s correlation.

Further evaluation of the correlation between *S1PR1* and immune cell populations by QUANTISEQ showed that *S1PR1* expression was positively correlated with B cells, macrophage (M1 and M2), and regulatory T cells in all databases (Figure 3). Interestingly, although not all correlations were significant, *S1PR1* was positively correlated with neutrophil infiltration in all three databases with better prognosis (GSE5287: ρ = 0.47, GSE13507: ρ = 0.16, GSE48075: ρ = 0.082). In contrast, *S1PR1* was negatively correlated with neutrophil (TCGA BLCA: ρ = −0.094, GSE32894: ρ = −0.047, GSE31684: ρ = −0.21) in the three databases where *S1PR1* was associated with poorer prognosis. This suggests that the enriched neutrophil infiltration may directly affect the prognosis prediction of bladder cancer patients using *S1PR1* expression.

### 3.3. Comprehensive Assessment of S1PR1 Expression Differences between Bladder Cancer Tumors and Normal Tissue

**Figure 4:**
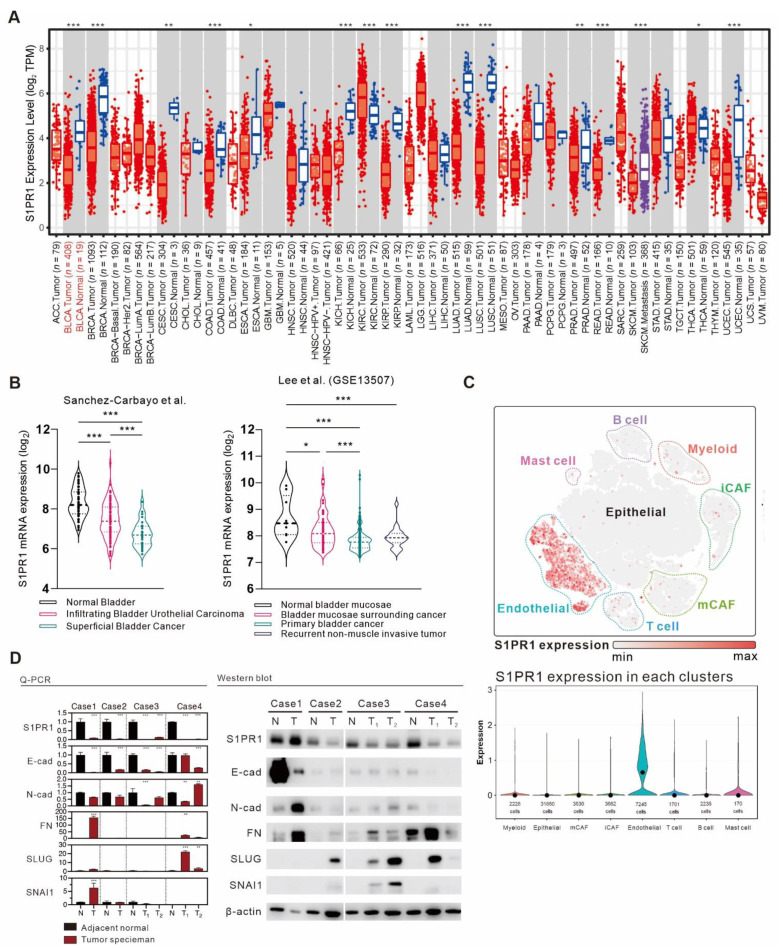
A comprehensive analysis of the differences in *S1PR1* expression in bladder cancer tumors versus normal tissues. (**A**) Differences in *S1PR1* expression in tumors and normal tissues among the 34 cancer types of TCGA adopted from TIMER 2.0 searching “*S1PR1*”. (**B**) Differences in *S1PR1* expression in normal versus clinically defined histopathological bladder cancer tissues from the bladder cancer database published by Sanchez-Carbayo et al. and Lee et al [31,32]. (GSE13507). (**C**) Bladder urothelial carcinoma single-cell RNA sequencing database adopted from Chen et al. showed that *S1PR1* was mostly expressed in endothelial cells, followed by immune cells. (**D**) mRNA and protein expression of *S1PR1* and EMT marker in four bladder cancer tumors and adjacent normal tissues. Student’s t-test or one-way ANOVA was utilized to analyze the statistical significance of the differences in *S1PR1* expression between groups. * *p* < 0.05, ** *p* < 0.01, *** *p* < 0.001. (N: adjacent normal tissue, T: tumor tissue, T1/2: samples from two separated tumors tissues, E-cad: E-cadherin, N-cad: N-cadherin, FN: Fibronectin).

Given the negative correlation between *S1PR1* expression and estimated tumor purity, we suggest that the proportion of normal tissue adulterated in tumor samples may also affect *S1PR1* expression. To clarify this issue, we attempted to identify the differential expression of *S1PR1* in normal tissue and bladder cancer tumor tissue. Gene expression analysis of the TCGA database provided by Timer 2.0 [20] showed that *S1PR1* expression was significantly higher in normal tissue than in tumor tissue in most cancers, including BLCA (Figure 4A). Further, the association of *S1PR1* expression differences with tissue types was evaluated in the clinical database of bladder cancer provided by Sanchez-Carbayo et al. and Lee et al. (Figure 4B) [35,36], showing that *S1PR1* expression was higher in normal bladder tissue and decreased as the tumor histology became more defined. For example, *S1PR1* expression was significantly lower in superficial bladder cancer or primary bladder cancer than in infiltrating bladder urothelial carcinoma or bladder mucosae surrounding cancer, suggesting that the actual expression of *S1PR1* may be affected when the tumor sample contains normal tissue. To further characterize the expression distribution of *S1PR1* in clinical bladder carcinoma samples, we analyzed *S1PR1* expression using the bladder urothelial carcinoma single-cell RNA sequencing database (Figure 4C) published by Chen et al. [21]. The results showed that *S1PR1* was mainly expressed in endothelial cells and to a lesser extent in various immune cells. In addition, endothelial cells were mainly found in normal and high-grade bladder urothelial carcinoma rather than low-grade (refer to Figure 1b in the publication of Chen et al., 2020), suggesting that high-grade tumors contain a high proportion of endothelial cells, which may be related to the high expression of endothelial and *S1PR1* due to tumor invasion of normal tissues.

We evaluated *S1PR1* expression in normal or tumor tissues from four clinical cases and analyzed the epithelial–mesenchymal transition (EMT) marker together, which is thought to be regulated by *S1PR1* expression, but with different effects in different tissues or spatial and temporal contexts [37,38]. The results showed that *S1PR1* mRNA and protein expression were mostly amplified in normal tissues, similar to E-cadherin expression, while N-cadherin, FN, SLUG, and SNAI1, which are mesenchymal markers, were higher in tumor tissues, suggesting a preliminary association between *S1PR1* expression and EMT in bladder cancer (Figure 4D).

### 3.4. Comparison of S1PR1 Expression in Bladder Cancer Cell Lines with Clinical Databases Reveals its Potential Function in Cell Adhesion

**Figure 5:**
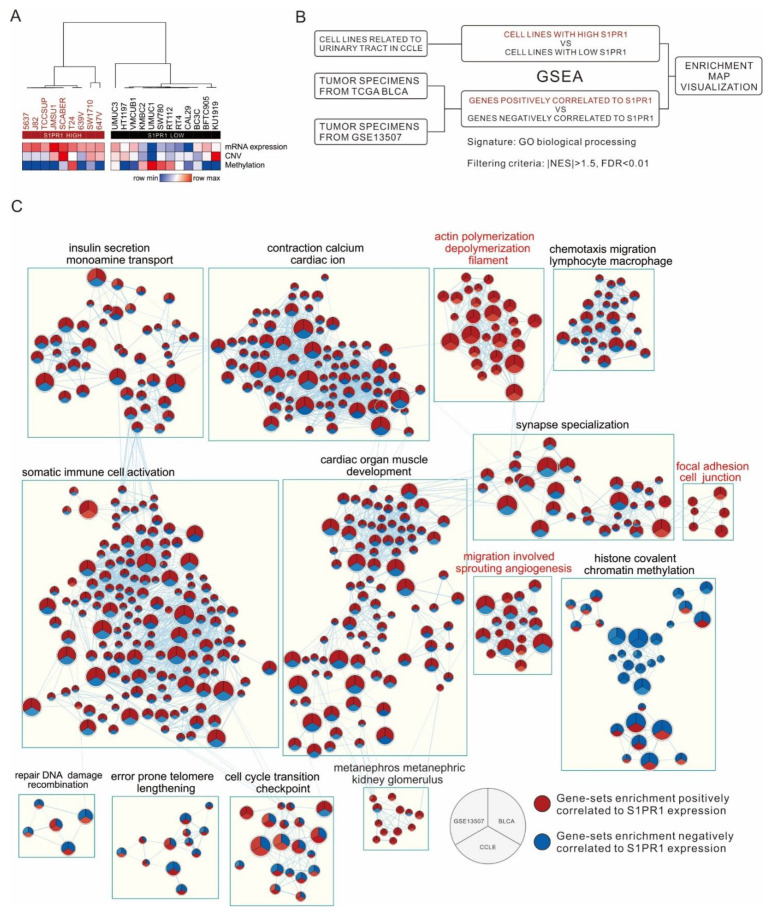
Investigation of *S1PR1* expression in bladder cancer cells for authentic biological response enrichment associations. (**A**) Heat map demonstrating *S1PR1* mRNA expression (fragments per kilobase per million, FPKM), copy number variation (log value), and methylation (β value) of 21 uroepithelial carcinomas in the CCLE database with unsupervised hierarchical clustering. (**B**) Flow chart presenting comparison of *S1PR1* expression and biological response association in CCLE bladder cancer cell lines or clinical bladder cancer tumors (TCGA BLCA, GSE13057). (**C**) Enrichment map visualization showing the GSEA scores of gene-sets significantly enriched in (**B**). The color represents the degree of normalized enrichment score (NES); red means the gene-sets are enriched in high *S1PR1* samples (NES > 1.5) and blue means the gene-sets are enriched in low *S1PR1* samples (NES < −1.5). All presented enriched gene sets have passed the screening criteria of *p*-value < 0.05, FDR < 0.01. Gene-sets without edge linkage were excluded to increase the ease of visualization of the results.

There are many factors in clinical bulk tissue that may affect *S1PR1* expression, such as the infiltration of endothelial or immune cells, which may lead to misinterpretation of the biological response of *S1PR1* expression in bladder cancer cells. To understand the direct effect of *S1PR1* expression on bladder cancer cells, 21 bladder cancer-related cell lines were screened from the CCLE database for *S1PR1* expression analysis, showing that the high *S1PR1* expression group generally had lower methylation and the low *S1PR1* expression group had higher methylation in addition to lower copy number variation (Figure 5A). Further, GSEA was performed after clustering cell lines with high or low *S1PR1* expression. On the other hand, GSEA was performed with *S1PR1* expression-related genes in TCGA BLCA (high *S1PR1* associated with poor prognosis) and GSE13507 (low *S1PR1* associated with poor prognosis) (illustrated as Figure 5B), and the results of the three GSEAs were visualized using the Enrichment map in Cytoscape (Figure 5C). The results showed that the node cluster associated with immune cell activation in the clinical database was not enriched in the cell lines, suggesting that the biological response associated with *S1PR1* expression in clinical tissues is indeed influenced by the immune microenvironment. On the contrary, we observed that angiogenesis and cell adhesion gene clusters were positively associated with *S1PR1* expression in all three, suggesting that the real effect of *S1PR1* on bladder cancer is related to these gene clusters.

### 3.5. S1PR1 Expression Shows an Opposite Association with the Promoting of Epithelial–Mesenchymal Transition

**Figure 6:**
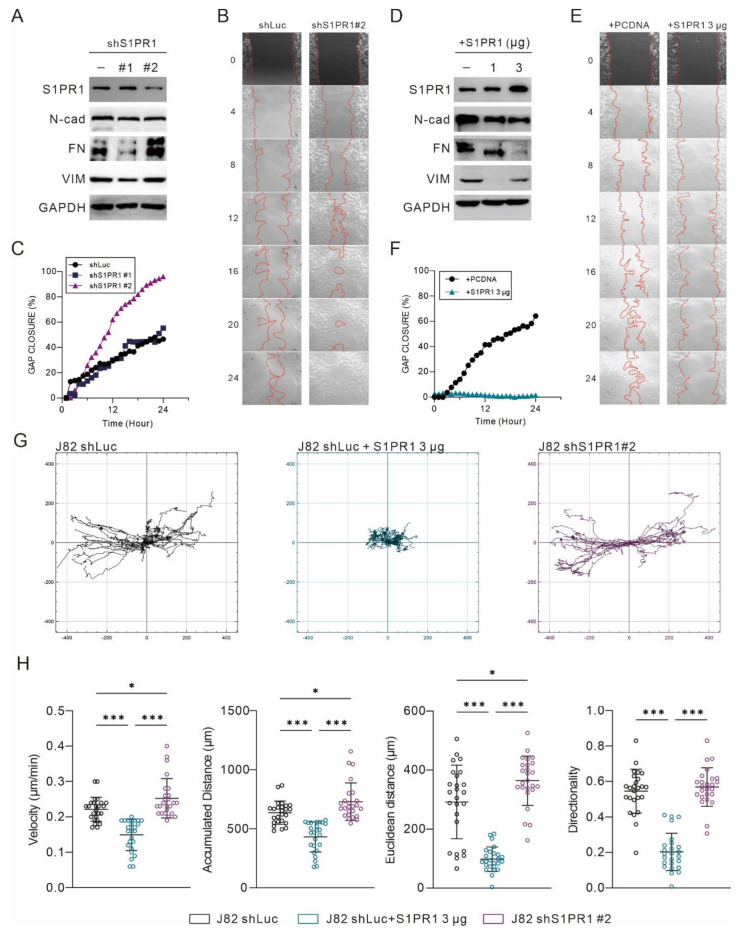
Negative correlation between *S1PR1* expression and bladder cancer cell motility. (**A**,**D**) Western blot demonstrating the efficacy of *S1PR1* expression manipulation and matching EMT marker expression. (**B**,**E**) Twenty-four hour live cell image tracking showed the progressions in gap area at multiple time points. (**C**,**F**) Gap closure (%) presents the effect of manipulating *S1PR1* expression on wound healing. (**G**) Migration tracking plot for J82 shLuc, shLuc + *S1PR1* (3 μg), and J82 sh*S1PR1*#2. Cell migration was tracked for 48 h after the wound healing assay started, with cell positions determined every 30 min. In each panel, the center indicates the starting point. (**H**) Statistical analysis of the J82 tracking cell migration rate, distance, and directionality, with lines showing mean and standard deviation. J82 shLuc (n = 29); J82 + *S1PR1* 3 μg (n = 30); J82 sh*S1PR1*#2 (n = 30). * *p* < 0.05, *** *p* < 0.001. (VIM: vimentin, GAPDH: glyceraldehyde 3 phosphate dehydrogenase) (for uncropped Western Blot images, please refer to Supplementary File S1).

Among the 21 uroepithelial cancer cell lines, only four cell lines, T24, J82, JMSU1, and SCABER, expressed high amounts of **S1PR1**, and its mRNA expression was not even detected in most cell lines. The expression of **S1PR1** was positively correlated with the variation of copy number, especially in JMSU1 and SCABER, which had a high copy number of the **S1PR1** gene (Figure 4A). Only the J82 bladder cancer cell line has a near normal copy number and moderate mRNA expression of the **S1PR1** gene. Therefore, to avoid potential interference with genetic abnormalities, the J82 bladder cancer cell line was used to establish a stable expression of **S1PR1** targeting shRNA clone (Figure 6A–C). In addition, the control cells (J82 shLuc) were transfected with pcDNA3.1-*S1PR1* plasmids for overexpression (Figure 6D–F).

Confirming the successful manipulation of *S1PR1* expression in J82 cells, it was found that *S1PR1* inhibition was associated with the enhancement of EMT (Figure 6A), while *S1PR1* over-expression inhibited EMT (Figure 6D). A further 24 h live cell imaging showed that the rate of gap healing was significantly increased when *S1PR1* expression was inhibited (Figure 6B). In particular, there was a significant upregulation in the clone that significantly inhibited *S1PR1* expression (sh*S1PR1*#2) (Figure 6C). In contrast, the rate of gap healing was significantly reduced upon overexpression of *S1PR1* (3 μg) (Figure 6E,F). As assessed by the cell migration trajectory (Figure 6G), overexpression of *S1PR1* significantly inhibited the migration distance of J82. Further evaluation of velocity, accumulated distance, Euclidean distance, and directionality showed that the over-expression of *S1PR1* significantly inhibited cell mobility, suggesting that *S1PR1* may affect cell movement by modulating cell adhesion (Figure 6H).

### 3.6. The Administration of FTY-720 Promotes EMT in Bladder Carcinoma

**Figure 7:**
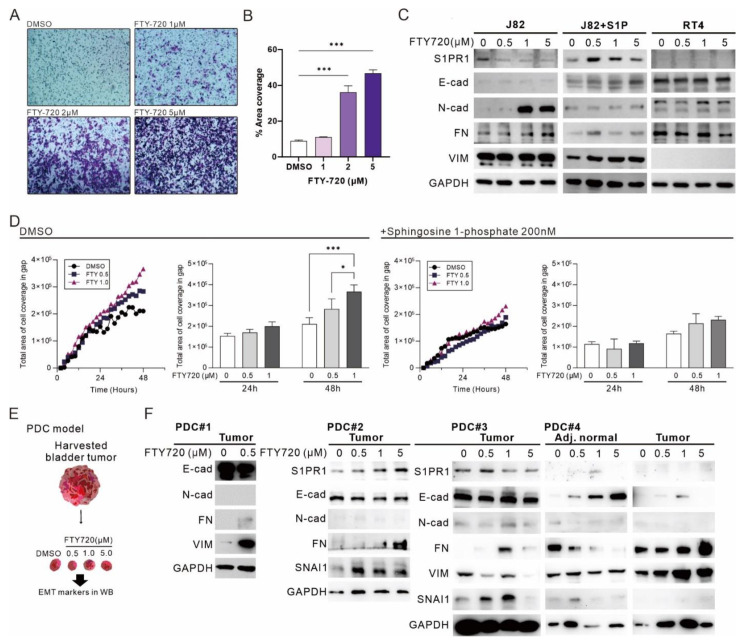
The administration of FTY-720 promotes EMT in bladder carcinoma. (**A**) Transwell migration assay showing the effect of FTY-720 treatment for 24 h (×100). (**B**) Area coverage (%) was calculated by quantifying the ratio of cell coverage per field using ImageJ. (**C**) Western blot showed the expression of *S1PR1*- and EMT-related markers after different dosage treatment of FTY-720 in J82 and RT4. Sphingosine 1-phosphate (200 nM) was added at the same time of FTY-720 treatment. (**D**) J82 cell gap coverage curves treated with FTY-720 in the presence and absence of S1P and statistical analysis of the number of cells in the gap at 24 h and 48 h. The curve data are presented as mean values only to reduce reading interference. (**E**) Illustration of patient-derived tumor culture (PDC) model. (**F**) Western blot showed four sets of clinical bladder cancer tumors with *S1PR1*- and EMT-related marker expression measured 48 h after FTY-720 addition. Bar charts are presented as the mean ± SD based on three independent experiments. * *p* < 0.05, *** *p* < 0.001 (for uncropped Western Blot images, please refer to Supplementary File S1).

FTY-720 is identified to inhibit cell proliferation and promote apoptosis by regulating *S1PR1*; however, the effect of FTY-720 on EMT in bladder cancer remains unknown. Transwell migration assay showed that the metastatic capacity of J82 increased with increasing FTY-720 treatment dose (Figure 7A), and the total cell coverage area was significantly increased with 2 and 5 μM FTY-720 treatment (Figure 7B). Similarly, FTY720 treatment inhibited E-cadherin expression and promoted mesenchymal marker expression, a phenomenon that was disturbed by the addition of S1P, but not in RT4 cells that did not express *S1PR1* (Figure 7C). In the wound healing assay, treatment with FTY720 promoted gap closure, especially at 48 h, with a significant difference. In contrast, FTY720 treatment in the presence of S1P had no significant effect on gap closure (Figure 7D), suggesting that inhibition of *S1PR1* by FTY720 promoted EMT in bladder cancer cells. Finally, to understand the overall effect of FTY720 treatment on human bladder cancer tumors, we established a patient-derived tumor culture model [29] (Figure 7E). A decrease in E-cadherin and increase in mesenchymal marker due to FTY720 treatment was observed in all four cases (Figure 7F), suggesting that bladder cancer tumors and cell lines respond similarly to FTY-720 treatment. In particular, in the case of PDC#4, the response to FTY720 in adjacent normal tissues and bladder cancer tumors showed an opposite trend, implying that FTY-720 may have divergent responses in different types of tissues or cancers.

## 4. Discussion

The physiological function and importance of *S1PR1* as a G protein-coupled receptor in vascular endothelial cells have been well established [39,40,41,42]. In the immune system, the expression of *S1PR1* is associated with selective in vivo recruitment, egress, and activation of various immune cells [10,43,44,45,46,47,48,49,50]. However, the role of *S1PR1* in cancer remains controversial. Correlation of *S1PR1* with pathological grade in tumors suggests its potential as a prognostic tool for patients with bladder cancer as well as liver and gallbladder cancers [37,51,52,53]. On the other hand, low *S1PR1* expression is suggested to be linked to poor prognosis in breast and lung cancer [54,55]. Given the complexity of immune cell infiltration and tumor purity, the exact expression and inhibitory effect of *S1PR1* in bulky bladder cancer tumor still needs to be validated.

In this study, we collected six databases of urothelial carcinoma with accompanying survival status and follow-up time for the association between *S1PR1* expression and patient prognosis (Figure 1). Overall, high *S1PR1* expression was associated with poorer prognosis, but the prognosis of patient survival showed a divergent trend in individual databases. Analysis by tumor purity assessment showed that *S1PR1* expression was negatively correlated with tumor purity in most databases (Figure 2A), as observed by Zhong et al. in breast and lung cancer [55]. Further, in silico simulation of immune cell infiltration showed that **S1PR1** expression was generally positively correlated with B cells, macrophage, and regulatory T cells, suggesting that assessment of immune cell infiltration may help to clarify the source or function of **S1PR1** expression in tumor tissues (Figure 2B) [56,57]. For instance, Liu et al. reported the correlation between *S1PR1* expression in bladder cancer cells convening regulatory T cells and poor prognosis [15]. In addition, **S1PR1** expression was positively correlated with neutrophil infiltration in three databases, where *S1PR1* was associated with better prognosis, suggesting that better prognosis may due to higher neutrophil infiltration [58,59], implying that *S1PR1* expression may be susceptible to the degree of immune cell infiltration in the tumor microenvironment.

The TCGA database shows that *S1PR1* expression is significantly higher in normal tissues than in tumor tissues for most cancers (Figure 3A). The work of Sanchez-Carbayo et al. and Lee et al. showed that *S1PR1* expression was reduced at low grade tumor (Figure 3B). Further, the single-cell RNA sequencing results reported by Chen et al. showed that most *S1PR1* expression was from endothelial cells and a few from multiple immune cells, both of which were underrepresented in low grade tumor (Figure 3C). These results suggest that the expression of *S1PR1* might be deeply affected by the purity of tumor cell composition in the lesioned tissue. Samples collected from high-grade tumor cells are more likely to be adulterated with normal tissue, which may explain the association of high *S1PR1* expression with poor prognosis observed in some databases. Nevertheless, whether *S1PR1* expression in tumor cell may affect the generation of tumor-associated endothelial cells requires further investigation. As our analysis shows that *S1PR1* expression is associated with angiogenesis (Figure 3C), it is possible that bladder cancer cells overexpressing *S1PR1* may affect tumor progression by altering microenvironmental angiogenesis [50,56,57]. When *S1PR1* expression was manipulated in J82, it was shown that overexpression of *S1PR1* had an inhibitory effect on bladder cancer cell migration, possibly associated with enhanced cellular apposition, echoing the analysis in Figure 3C. Conversely, shRNA interference with *S1PR1* expression or inhibition of *S1PR1* by FTY-720 accelerated bladder cancer cell migration, and the addition of S1P antagonized the effect of FTY-720. Moreover, a similar phenomenon could be observed in the patient-derived tumor primary culture model, suggesting that clinical inhibition of *S1PR1* may cause accelerated metastasis of bladder cancer cells.

High expression of *S1PR1* was significantly associated with poor prognosis in multiple cancer databases, raising the possibility of its potential role in promoting tumorigenesis. Based on this inference, FTY-720 has been reported and demonstrated to induce apoptosis in a variety of cancer cells, including bladder cancer [18,60]. Moreover, the mechanism of EMT inhibition by FTY-720 in cholangiocarcinoma and glioblastoma has also been proposed [61,62]. However, our study clearly indicates that reduction of *S1PR1* expression by human manipulation may cause a promotion in EMT in bladder cancer cells and patient tumor tissue, a phenomenon consistent with FTY-720 treatment. Different responses to FTY-720 in normal or tumor tissues also indicate divergences in response between various types of tissues or cancers; this may be due to the fact that normal tissue is usually rich in endothelium [63,64]. Although a majority of the literature has confirmed the induction of apoptosis in cancer cells by FTY-720, we still need to pay attention to its potential role in EMT induction. Furthermore, whether the apoptosis induction caused by FTY-720 is related to the EMT-induced anoikis needs to be further clarified. The inhibition of *S1PR1* contributes to the reduced interaction with the ECM, thus allowing a higher migration ability. Cell proliferation may be reduced as a result and apoptosis may occur owing to separation from the matrix [65]. Furthermore, cancer cells may thus develop an anti-anoikis mechanism and become more resistant to chemotherapy or accelerate the progression of metastasis [66,67,68].

## 5. Conclusions

In conclusion, in general, high *S1PR1* expression is associated with poor prognosis, but this observation may be interfered with by endothelial or immune cell infiltration, so the accuracy of *S1PR1* expression for clinical diagnosis needs to be further evaluated. *S1PR1* expression promotes cancer cell adhesion and, conversely, inhibition of *S1PR1* by genetic manipulation or FTY-720 may increase bladder cancer cell migration ability. Although it is known that inhibition of *S1PR1* has multiple mechanisms to counteract tumor growth, the resulting risk of metastasis should not be overlooked. Therefore, the use of FTY-720 as a concurrent treatment strategy for bladder cancer requires further evaluation and observation.

## Figures and Tables

**Table 1:** Primer sequences used in the qPCR assay.

Gene	Forward Primer	Reverse Primer
*SLUG*	AAGCATTTCAACGCCTCCAAA	GGATCTCTGGTTGTGGTATGACA
*CDH1*	GCCTCCTGAAAAGAGAGTGGAAG	TGGCAGTGTCTCTCCAAATCCG
*GAPDH*	CATCACTGCCACCCAGAAGACTG	ATGCCAGTGAGCTTCCCGTTCAG
*CDH2*	TGCGGTACAGTGTAACTGGG	GAAACCGGGCTATCTGCTCG
*SNAI1*	TCGGAAGCCTAACTACAGCGA	AGATGAGCATTGGCAGCGAG
*FN1*	CGGTGGCTGTCAGTCAAAG	AAACCTCGGCTTCCTCCATAA
**S1PR1**	ATCATGGGCTGGAACTGCATCA	CGAGTCCTGACCAAGGAGTAGAT

## Data Availability

GSE5287, GSE13507, GSE19915, GSE31684, and GSE32894 whole gene expression and clinical data used in this study were obtained from NCBI Gene Expression Omnibus. TCGA BLCA and CCLE whole gene expression and corresponding data were obtained from UCSC Xena (https://xenabrowser.net/datapages/, accessed on 26 March 2021). The whole gene expression databases of Sanchez-Carbayo et al. and Chen et al. are available from the supplemental data of the authors’ publications.

## Supplementary Materials

The following are available online at https://www.mdpi.com/article/10.3390/cancers13174474/s1, File S1: Uncropped Western blot figures.
