# Supplementary figures and images for: Targeting S1PR1 May Result in Enhanced Migration of Cancer Cells in Bladder Carcinoma
Source: Cancers (Basel). 2021 Sep 5;13(17):4474. doi: 10.3390/cancers13174474 (PMC8431630; doi:10.3390/cancers13174474)

C

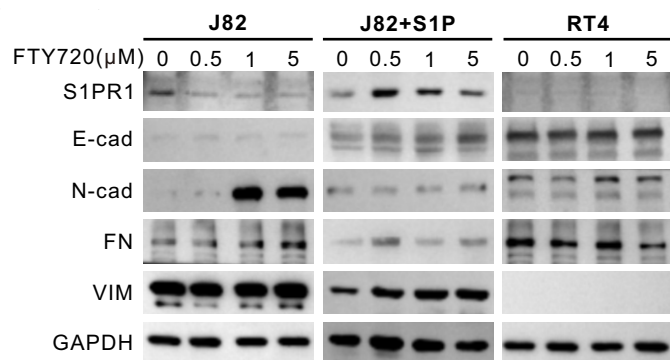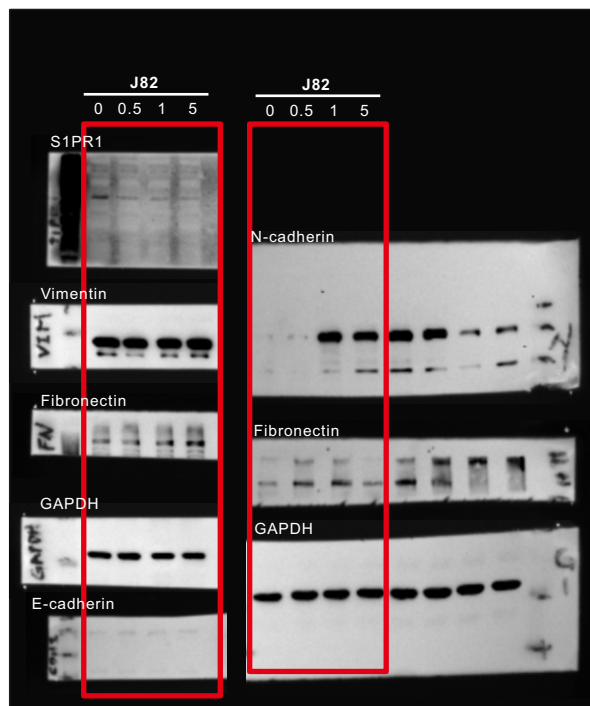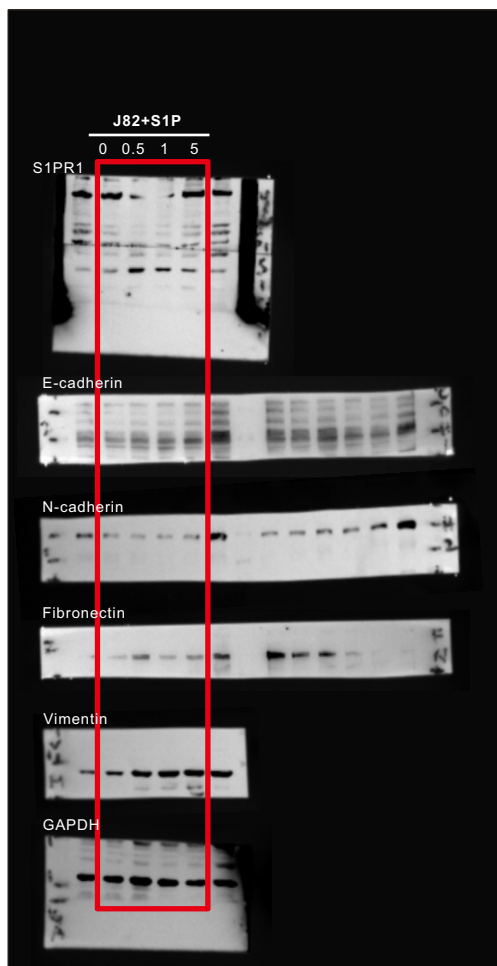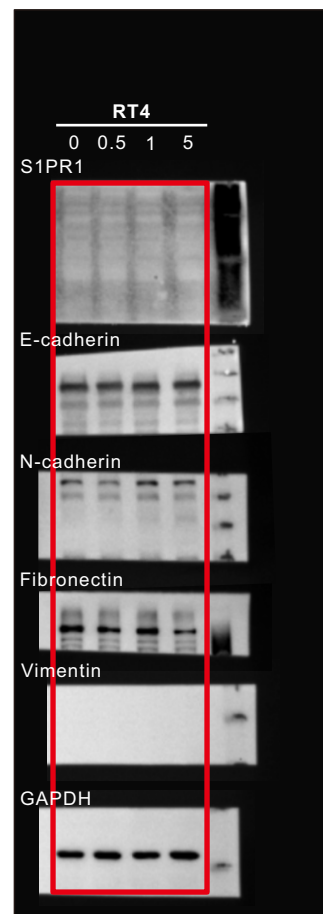

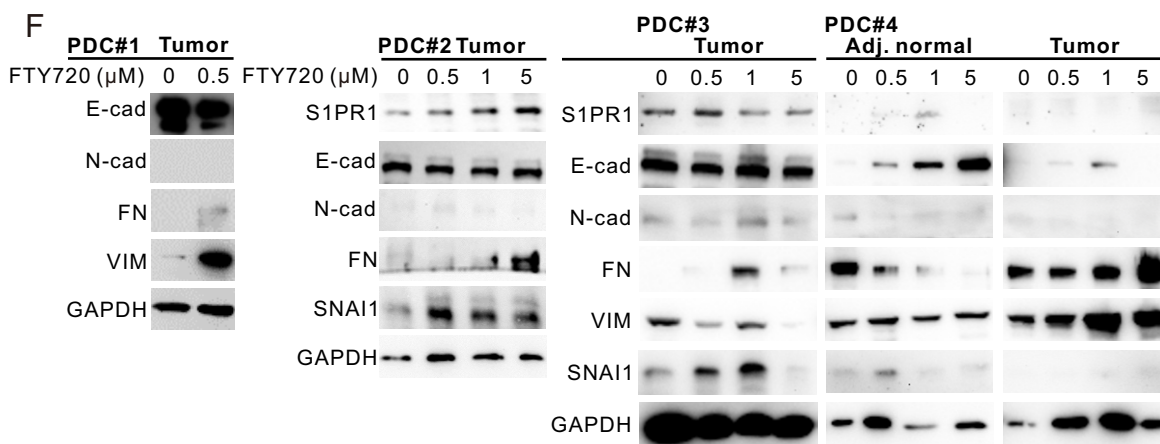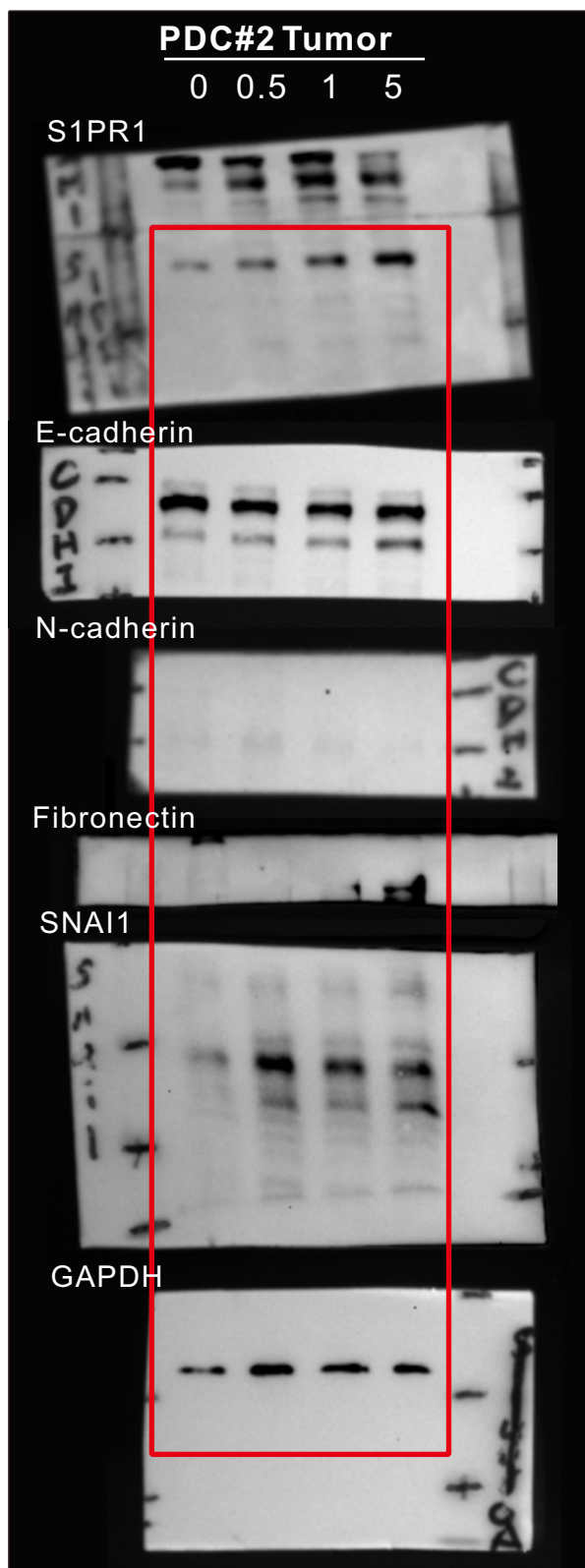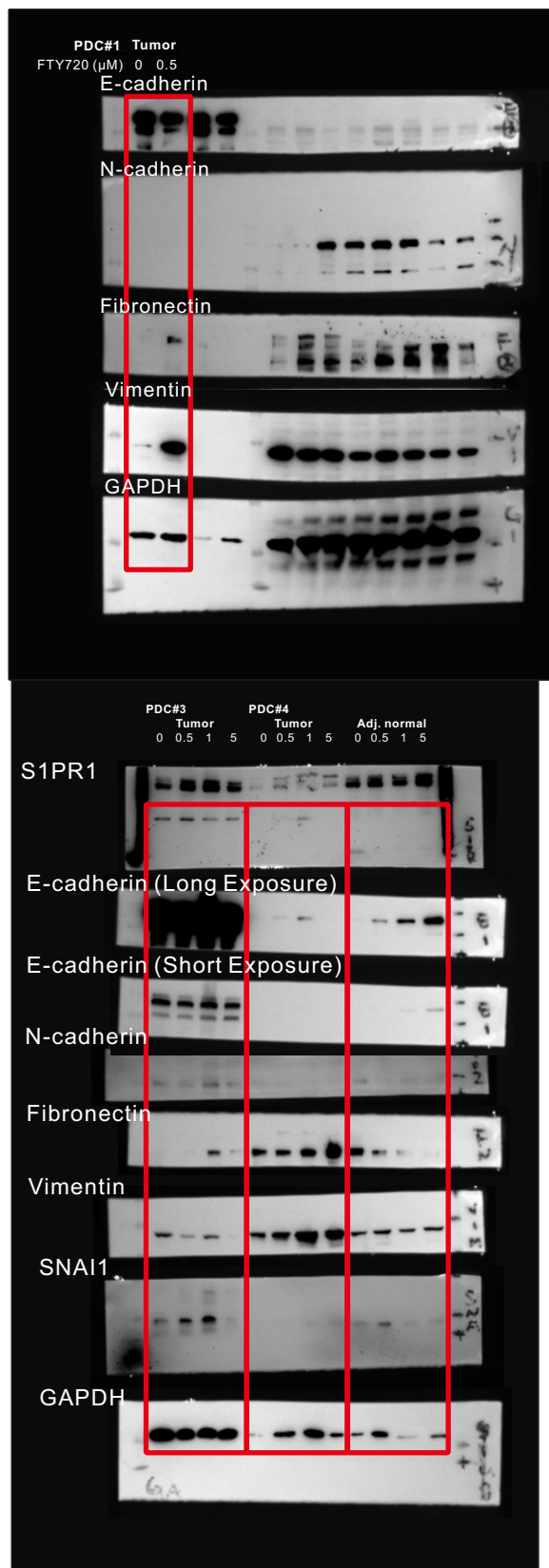

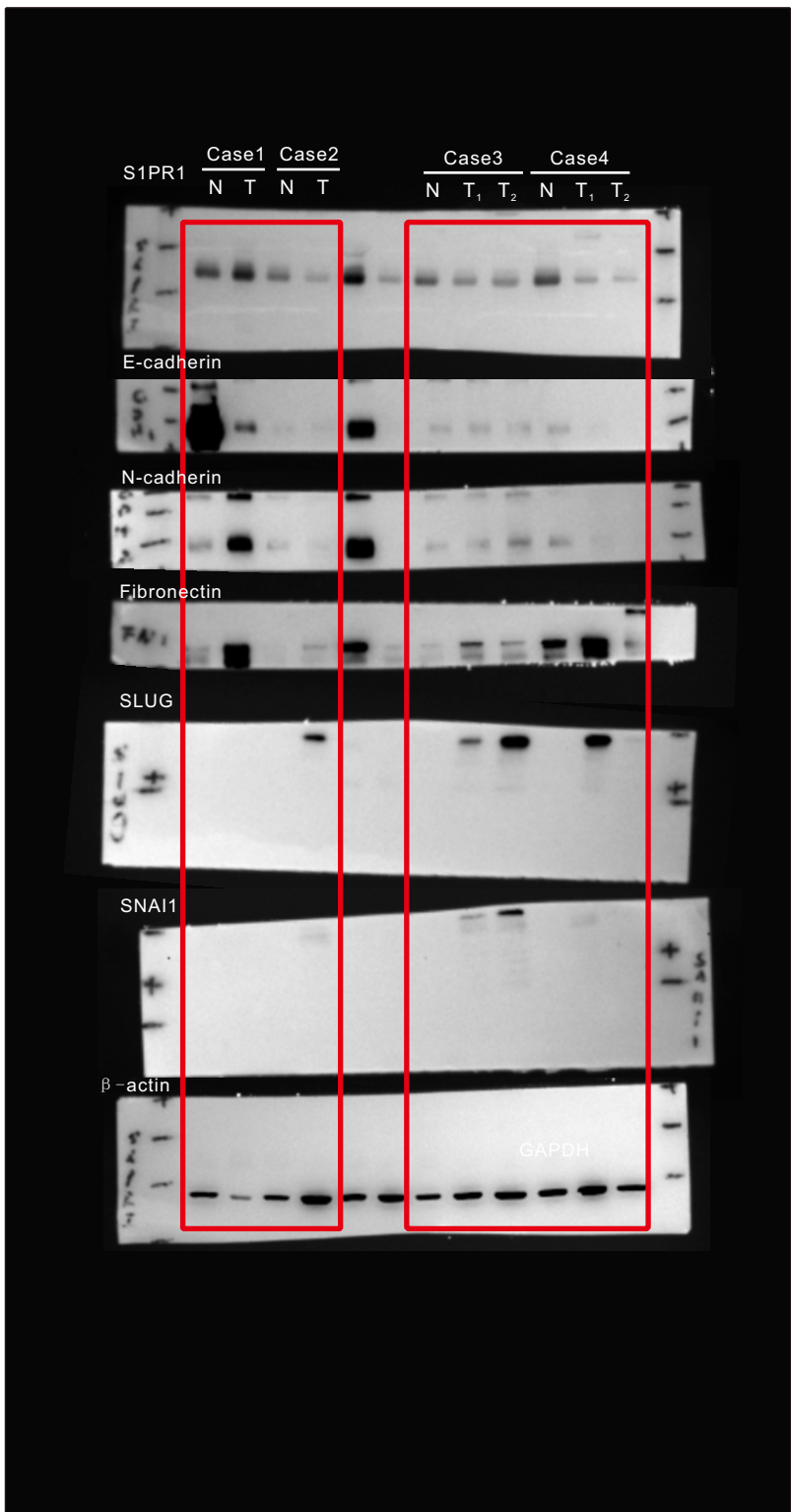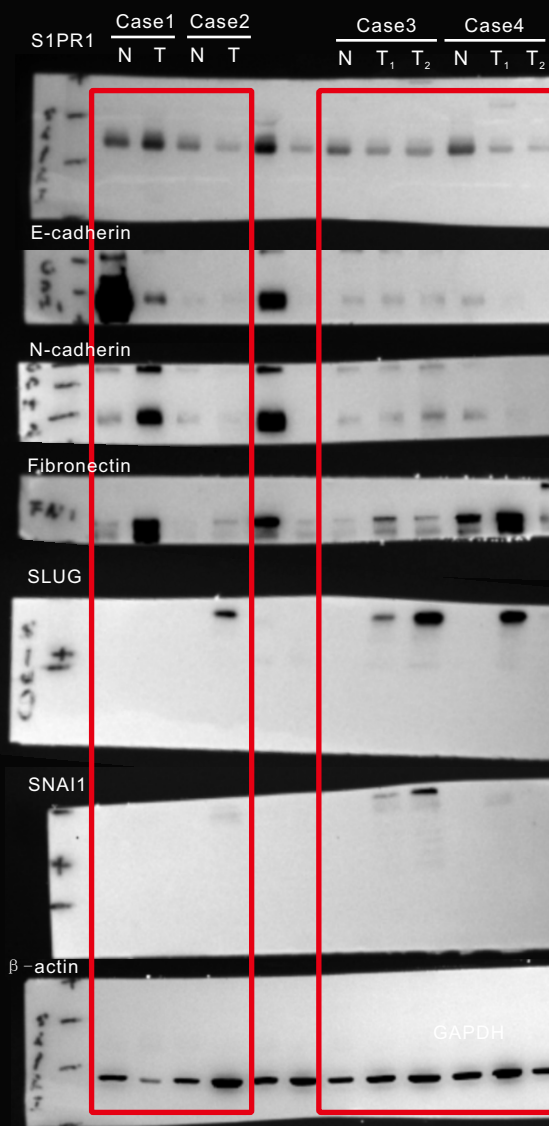

A

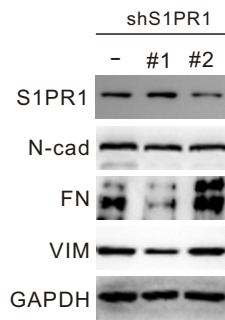

D

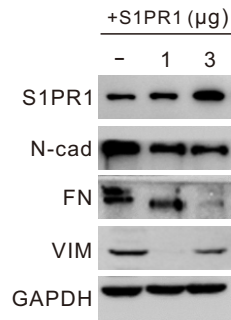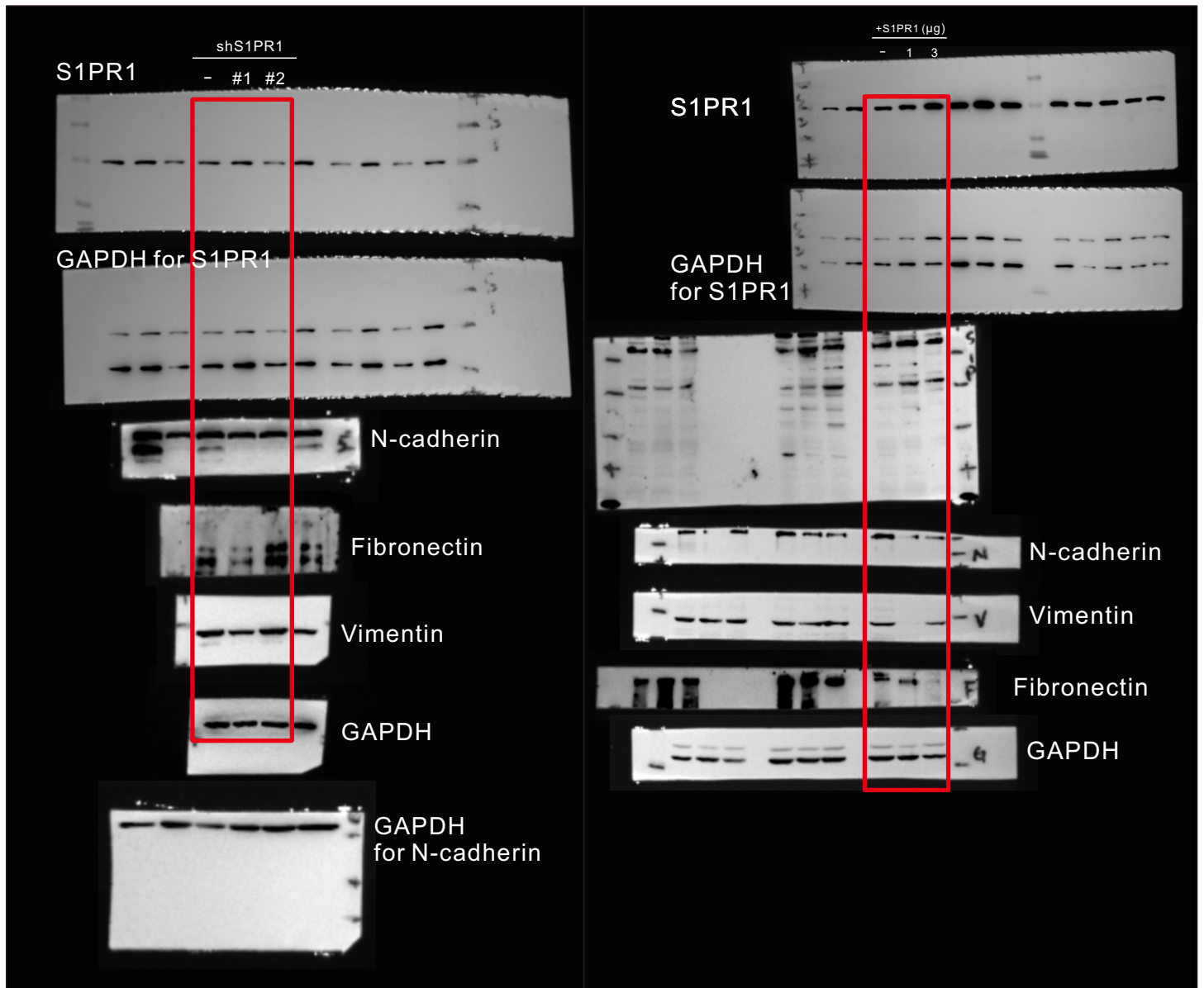

Supplement: Supplementary file 1 [file cancers-13-04474-s001.zip › cancers-1322704-supplementary.pdf]
